# High Device Performances and Noise Characteristics of AlGaN/GaN HEMTs Using In Situ SiCN and SiN Cap Layer

**DOI:** 10.3390/nano12040643

**Published:** 2022-02-14

**Authors:** Ki-Sik Im, Siva Pratap Reddy Mallem, Jin-Seok Choi, Young-Min Hwang, Jae-Seung Roh, Sung-Jin An, Jae-Hoon Lee

**Affiliations:** 1 Advanced Material Research Center, Kumoh National Institute of Technology, Gumi 39177, Korea; hdhym@kumoh.ac.kr; 2 School of Materials Science and Engineering, Kyungpook National University, Daegu 41566, Korea; dr.mspreddy@gmail.com; 3 Department of Advanced Materials Science and Engineering, Kumoh National Institute of Technology, Gumi 39177, Korea; choijs1220@kumoh.ac.kr (J.-S.C.); jsroh@kumoh.ac.kr (J.-S.R.); sungjinan@kumoh.ac.kr (S.-J.A.); 4 Yield Enhancement Team, Foundry, Samsung Electronics Company Ltd., Pyeongtaek 17789, Korea; jaehoon03.lee@samsung.com

**Keywords:** AlGaN/GaN HEMT, in situ cap layer, low-frequency noise, pulse measurement

## Abstract

We fabricated and characterized AlGaN/GaN high-electron mobility transistors (HEMTs) with a nano-sized in situ cap layer (one is a silicon carbon nitride (SiCN) layer, and the other is a silicon nitride (SiN) layer) comparing to the conventional device without an in situ cap layer. The pulse characteristics and noise behaviors for two devices with in situ cap layers are much superior to those of the reference device without a cap layer, which means that the in situ cap layer effectively passivates the AlGaN surface. On the other hand, the device with an in situ SiCN cap layer showed the excellent device characteristics and noise performances compared to the other devices because of the reduced positive ionic charges and enhanced surface morphology caused by carbon (C) surfactant atoms during the growth of the SiCN cap layer. These results indicate that the AlGaN/GaN HEMT with the in situ SiCN cap layer is very promising for the next high-power device by replacing the conventional HEMT.

## 1. Introduction

AlGaN/GaN heterostructure exhibits the possibility for applications in high-power and high-frequency electronics [1,2]. The large conduction band discontinuity and the polarization effects formed at the AlGaN/GaN heterostructure offer large two-dimensional electron gas (2DEG) densities as well as high electron mobility. In addition, GaN and its alloy have a great opportunity in view of superior material properties, such as wide energy bandgap and large critical electric field. Therefore, these advantages allow them to obtain high current density with large breakdown voltage (V_br_), which is very essential for high output power.

In spite of the GaN-based material advantages and the mature device processing, AlGaN/GaN high-electron mobility transistors (HEMTs) have encountered several technical challenges [3,4]. One of the major issues is the current collapse phenomenon in AlGaN/GaN-based HEMTs caused by the surface trapping effect in the AlGaN barrier layer, which is adversely affecting the reliability of the device. The current collapse phenomenon is mainly due to the electron trapping in surface states on devices. Trapped electrons deplete the 2DEG densities in AlGaN/GaN heterostructure and thus deteriorate the current density as well as the dynamic on-resistance (R_on_) in AlGaN/GaN-based devices.

Surface passivation of AlGaN/GaN heterostructure effectively eliminates surface trapping effects by prohibiting Ga-O bonding of the AlGaN surface. Currently, Al_2_O_3_, SiO_2_, and Si_3_N_4_ deposited by atomic layer deposition (ALD) or plasma-enhanced chemical vapor deposition (PECVD) have been used for the surface passivation layer and/or gate dielectric layer [5]. This ex situ deposition method is easy to deposit and passivate the AlGaN surface. However, the AlGaN surface could be damaged or contaminated by various chemical solutions before the deposition of the dielectric layer. This is because the surface passivation is conducted during the device fabrication or at the end of the device fabrication.

On the other hand, the dielectric layer deposited by the in situ method can be directly grown on the AlGaN barrier layer in a metalorganic chemical vapor deposition (MOCVD) without exposure to air ambient [6,7,8,9,10]. Hence, the in situ dielectric layer well protects the AlGaN surface without plasma/etching damages or process-induced contaminations on the AlGaN surface during the device fabrication. As a result, the in situ dielectric layer can minimize the density of interface trap states from Ga-O bonds and suppress the current collapse.

Enhanced device performances with low interface trap density in the device with the in situ SiN cap layer have been investigated in previous literature [6,7]. Recently, several reports about in situ SiCN capped AlGaN/GaN devices have been conducted, which shows improved device performances as well as noise performances because the in situ SiCN cap layer effectively passivates the AlGaN surface layer [8,9,10]. However, there are no reports about the comparison of AlGaN/GaN HEMTs with in situ SiCN and SiN cap layers. In this work, the AlGaN/GaN HEMTs with SiCN and SiN cap layers have been fabricated and characterized compared to the conventional AlGaN/GaN HEMT without the cap layer as a reference device.

## 2. Materials and Methods

**Figure 1:**
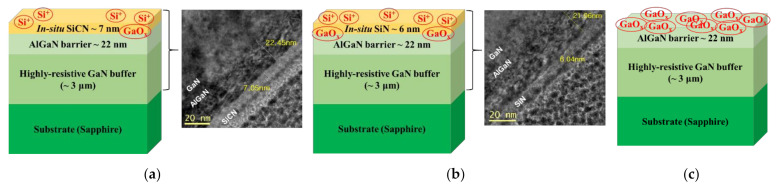
Schematic illustrations of the epitaxial structure and configuration for the fabricated devices and TEM images of (**a**) SiCN/AlGaN/GaN and (**b**) SiN/AlGaN/GaN heterostructure grown on sapphire substrate. Schematic image of (**c**) shows the reference device without in situ cap layer. The device with SiN cap layer contains many positive ionic charges (Si^+^ and GaO_x_), while the SiCN capped device has less positive ionic charges during the epitaxial growth. On the other hand, the reference device without in situ cap layers contains lots of the natural oxide (GaO_x_) on the AlGaN surface.

We demonstrate two different types of the in situ capped AlGaN/GaN HEMTs, one is with in situ SiCN layer, and the other is with in situ SiN layer. The AlGaN/GaN epitaxial structure with various in situ cap layers for the normally-on device operation are grown on 4-inch sapphire substrate by MOCVD. Figure 1 shows the cross-sectional epitaxial structures and their transmission electron microscope TEM (Thermo Fisher Scientific, Waltham, MA, USA) images of the AlGaN/GaN heterostructure with in situ SiCN and SiN cap layer. The epitaxial structures consist of 30 nm-thick initial nucleation GaN layer on the substrate, a 3 μm-thick semi-insulating GaN buffer layer, and a 22 nm-thick Al_0.12_Ga_0.88_N barrier layer. A 7 nm-thick SiCN cap layer is deposited using gas sources of Si (di-tertiary-butyl-silane, DTBSi), N (ammonia, NH_3_), and C (carbon tetrabromide, CBr_4_) in Figure 1a. For comparison, the AlGaN/GaN heterostructure with and without SiN cap layer were also grown. A 6 nm-thick SiN cap layer is grown using Si and N gas sources without C gas source, which is confirmed by the TEM image in Figure 1b.

Prior to device fabrication, the 2DEG properties of in situ SiCN/AlGaN/GaN heterostructure, measured by Hall measurements using the van der Pauw method, are a sheet resistance (R_sh_) of 1208 Ω/sq, electron mobility (μ_e_) of 1400 cm^2^/V·s, and electron concentration (n_s_) of 3.7 × 10^12^ cm^−2^. The R_sh_ of AlGaN/GaN heterostructure with and without SiN cap layer are obtained to be 1307 Ω/sq (μ_e_ of 1230 cm^2^/V·s and n_s_ of 3.9 × 10^12^ cm^−2^) and 1923 Ω/sq (μ_e_ of 1200 cm^2^/V·s and n_s_ of 2.7 × 10^12^ cm^−2^), respectively. The electrical properties for all devices are lower than those of the conventional AlGaN/GaN heterostructure (n_s_~1.0 × 10^13^ cm^−2^) because of the relatively low Al composition of 12% of AlGaN layer (the data are not shown). However, it is interesting that the overall 2DEG properties of the devices with in situ cap layer are higher than those of the device without in situ cap layer. The reason for the lowest n_s_ value of the no capped device is due to the acceptor-like surface state [8]. After growth of in situ cap layer, these acceptor-like surface states can be neutralized and induced the increased n_s_. On the other hand, the n_s_ value for the devices with in situ SiCN cap layer is slightly smaller than that of in situ SiN cap layer. This is believed to be due to the reduced positive ionic charges (such as Si^+^ and GaO_x_) [8,11,12] during the growth of SiCN cap layer using C surfactant atoms in CBr_4_ gas. On the other hand, the in situ growth of SiN cap layer without C surfactant increases the diffusion of Si atoms into the AlGaN layer.

Mesa isolation with 500 nm depth is performed by using the inductively coupled plasma reactive ion etching (ICP-RIE) with Cl_2_ gas. Ohmic metal stack (Si/Ti/Al/Ni/Au = 1/25/160/40/100 nm) is deposited on source and drain region by electron-beam evaporator and followed by two-step rapid thermal annealing (RTA) at 500 °C for 20 s plus 850 °C for 30 s in N_2_ ambient. Finally, metal stack (Ni/Au = 30/200 nm) is deposited for gate and pad metallization.

## 3. Results

### 3.1. I–V Characteristics

**Figure 2:**
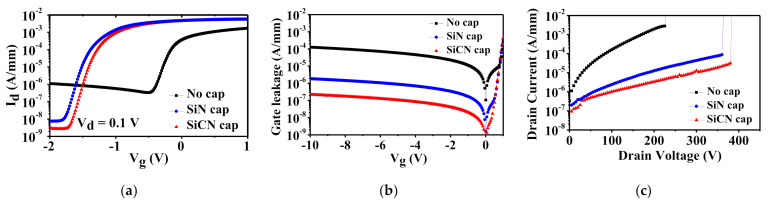
(**a**) I_d_ − V_g_ curves at V_d_ = 0.1 V and (**b**) I_g_ − V_g_ curves for all fabricated AlGaN/GaN HEMTs with and without cap layer. (**c**) Off-state breakdown voltage (V_br_) characteristics of measured devices with different kinds of cap layer at L_gd_ = 5 μm.

The DC characteristics of the fabricated normally-on AlGaN/GaN HEMTs with in situ SiCN/SiN cap layer at gate width (W_g_) = 50 μm, gate length (L_g_) = 5 μm, and a distance of gate-to-drain (L_gd_) = 4 μm are investigated in Figure 2. Figure 2a shows the I_d_ − V_g_ characteristics at linear region (V_d_ = 0.1 V). The AlGaN/GaN HEMTs with in situ SiCN and SiN cap layer exhibits the threshold voltage (V_th_) of −1.5 and −1.6 V, respectively, while the device without cap layer exhibits relatively high V_th_ of −0.3 V owing to no existence of cap layer under the gate region. The reason for the slightly positive V_th_ shift (~0.1 V) of the device with the SiCN cap layer is due to the decreased 2DEG density in order to satisfy the charge neutrality [8], which is well matched with the results of Hall measurements. This reflects that the positive ionic charges during the growth of the SiCN cap layer are less introduced compared to that of the SiN cap layer, as previously discussed. It is also noticed that the off-state leakage current of the device with the in situ SiCN cap layer is lowest compared to those of other devices. This means that the in situ SiCN cap layer effectively passivates the AlGaN surface layer, which reduces the surface leakage current at off-state.

**Figure 3:**
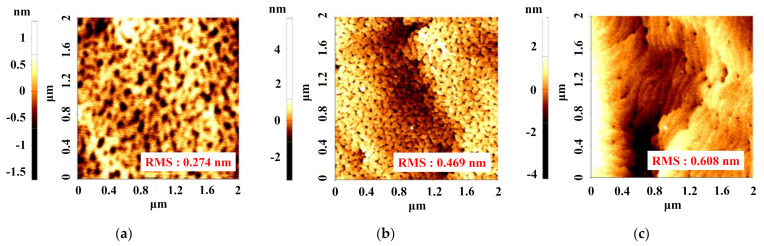
AFM images of AlGaN/GaN HEMTs with (**a**) SiCN and (**b**) SiN cap layer compared to (**c**) without cap layer including the root mean square (RMS) roughness values on 2 × 2 μm scan area.

To further examine the surface leakage current, the gate leakage currents for all devices are measured (Figure 2b). From the results of gate leakage measurement, it is clearly observed that the in situ SiCN capped device exhibits the lowest gate leakage current due to the good insulator quality. This is also because of the improved surface morphology, which is confirmed by atomic force measurement AFM (Park Systems, Suwon, Korea) images in Figure 3. Surface morphology can be improved by surfactant atoms, which enhance the surface mobility during the growth of the in situ cap layer [13]. The CBr_4_ gas as a C source plays an important role of surfactant, which leads to the smooth surface roughness of the device with the in situ SiCN cap layer [8]. In addition, this C surfactant can probably mitigate the defect density from observing the reduced dark spots in AFM images of the in situ SiCN capped device when compared to those of the other devices (Figure 3). This also leads to the improved device performances.

The off-state breakdown characteristics of the fabricated devices with L_gd_ of 4 μm were investigated by utilizing semiconductor analyzers (Keithley model 2410 high voltage supplier (Keithley, Cleveland, OH, USA) and Agilent B1500A (Keysight, Santa Rosa, CA, USA), as shown in Figure 2c. When the drain current suddenly reaches over 10 mA/mm, the drain voltage is defined as V_br_. The devices with the in situ SiCN/SiN and without cap layer present V_br_ of 380, 360, and 230 V, respectively. The reason for the largest V_br_ of the device with the SiCN cap layer is because of the mitigated surface current leakage at high electric field, which is consistent with the results of the off-state leakage current and gate leakage current.

### 3.2. Surface Trapping Effect on Pulsed I_d_ − V_d_ Characteristics

**Figure 4:**
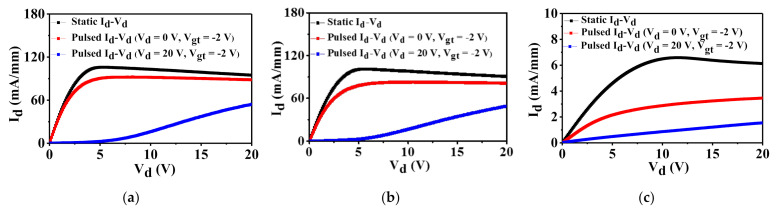
Static and pulsed I_d_−V_d_ characteristics for AlGaN/GaN HEMTs with (**a**) in situ SiCN cap layer, (**b**) in situ SiN cap layer, and (**c**) without cap layer at V_g_ = 1 V and V_d_ = 0~20 V.

Figure 4 compares the output (or static) I_d_−V_d_ characteristics (black curves) of AlGaN/GaN HEMTs with and without in situ cap layer at V_g_ = −2 V and sweeping V_d_ = 0~20 V. Both devices with cap layer in Figure 4a,b exhibit almost the same maximum drain current (I_d,max_) of 106 mA/mm. The on-resistance (R_on_) for both capped devices, extracted from the linear region at V_g_ where maximum I_d_ flows, is a relatively low 24 ohm-mm. On the other hand, the device without the in situ cap layer shows much lower I_d,max_ (6 mA/mm) and degraded R_on_ (703 ohm-mm) than those of the other devices in Figure 4c. This result is attributed to be due to the large access resistance in the no capped device and decreased 2DEG properties [8,9].

Pulsed I_d_−V_d_ characteristics of AlGaN/GaN HEMTs with and without cap layer are plotted in Figure 4 at V_g_ = 1 V with two quiescent bias points, [V_gt,Q_, V_d,Q_] = [−2 V, 0 V] (red curves) and [−2 V, 20 V] (blue curves) (the gate overdrive voltage, V_gt,Q_ = V_g,Q_ − V_th_). The pulse width and period are set to be 50 μs and 1 ms, respectively. When we compare the red and black curves, the devices with cap layer show small drain current degradation, which is named “gate lag” due to the electron trapping effect in the surface of the AlGaN barrier layer. The in situ SiCN capped device exhibits slightly smaller gate lag than that of the in situ SiN capped device. This is attributed to the smooth surface/interface states of the in situ SiCN cap layer. However, the device without the cap layer presents a severe current collapse phenomenon. On the other hand, the drain lag can be verified by comparing the pulsed I_d_−V_d_ with [V_gt,Q_, V_d,Q_] = [−2 V, 0 V] and [−2 V, 20 V] because of the electron trapping effect in the GaN buffer at high drain voltage. All devices show almost the same current collapse, which indicates that the fabricated devices show almost the same GaN buffer resistivity. Therefore, it is mentioned that the in situ cap layer effectively suppresses the surface trapping effects.

### 3.3. Noise Characteristics

**Figure 5:**
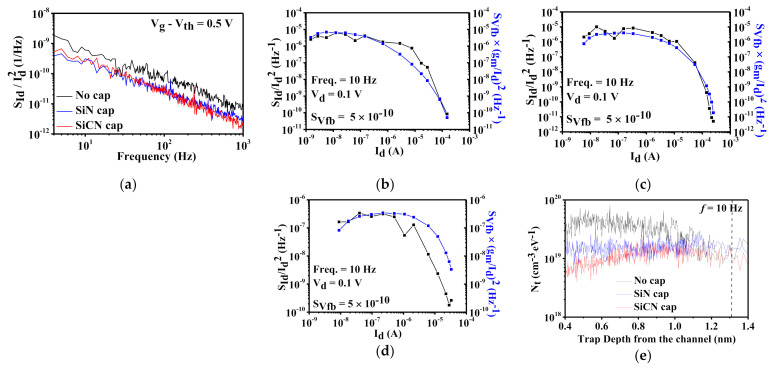
(**a**) Normalized noise spectral density (S_Id_/I_d_^2^) according to the f at V_g_ – V_th_ = 0.5 V and V_d_ = 0.1 V. S_Id_/I_d_^2^ (red circle) and (g_m_/I_d_)^2^ (blue line) versus I_d_ at V_d_ = 0.1 V and f = 10 Hz for AlGaN/GaN HEMTs with (**b**) in situ SiCN, (**c**) SiN, and (**d**) no cap layer. (**e**) Trap density (N_t_) versus trap depth (Z), taken from the noise spectrum at V_g_~V_th_ for three devices.

Noise characteristics can be used to evaluate the interface/surface traps in the gate oxide layer and find the conduction channel mechanism. The fabricated devices are measured by using a fully automatic system (Synergie-concept, NOISYS7) at room temperature [14]. The normalized drain-current noise spectral density (S_Id_/I_d_^2^) is obtained with biasing from subthreshold to strong accumulation region at V_d_ = 0.1 V according to the frequency from 4 to 10^4^ Hz, as shown in Figure 5a. In order to find the conduction mechanism in the channel layer, S_Id_/I_d_^2^ according to the drain current (I_d_) matches with (g_m_/I_d_)^2^. If S_Id_/I_d_^2^ is well proportional to (g_m_/I_d_)^2^, the carrier number fluctuations (CNF) noise model prevails, which explains that the electron trapping/detrapping between the oxide layer and the surface channel, as a following equation, [15,16],

(1) $$\frac{S_{\mathrm{Id}}}{I_{d}^{2}}={\left(\frac{g_{m}}{I_{d}}\right)}^{2}S_{\mathrm{Vfb}}$$

where

(2) $$S_{\mathrm{Vfb}}=\frac{q^{2}{\mathrm{kT}\lambda N}_{t}}{{\mathrm{WLC}}_{\mathrm{ox}}^{2}f}$$

is flat-band voltage fluctuations including q is the electron charge, kT is the thermal energy, λ is the oxide tunneling attenuation distance, N_t_ is the volumetric oxide trap density, WL is the channel area, C_ox_ is the gate dielectric capacitance per unit area, and f is frequency. When compared with noise data for in situ SiCN and SiN capped devices, S_Id_/I_d_^2^ of both devices are same with the dependence with (g_m_/I_d_)^2^ and the S_Vfb_ value of 5 × 10^−10^ V^2^·Hz^−1^ (the corresponding N_t_ is extracted to be 1.1 × 10^19^ cm^−3^·eV^−1^) (Figure 5b,c). The reason for the same noise characteristics regardless of the cap layer is because both in situ cap layers are well protected to the AlGaN surface region. On the other hand, the reference device without cap layer has relatively large discrepancy between S_Id_/I_d_^2^ and (g_m_/I_d_)^2^ above I_d_~5 × 10^−5^ A due to the degraded series on-resistance (Figure 5d) [17].

### 3.4. Depth Dependent Trap Density from 1/f Noise Curves

From 1/*f* noise spectrum, the trap depth (Z) can be determined by the following equation [18,19],

(3) $$Z=\frac{1}{\alpha_{t}}\ln\left(\frac{1}{2\pi f\tau_{0}}\right)\;$$

where

(4) $$\alpha_{t}=\frac{2}{\hbar }\sqrt{2{\mathrm{qm}}_{\mathrm{AlGaN}}\Phi_{\mathrm{it}}}{\;\mathrm{and}\;\tau }_{0}=\frac{1}{{n\sigma \nu }_{\mathrm{th}}}$$

α_t_ is the oxide tunneling attenuation factor, ℏ is the Planck constant, m_AlGaN_ is the tunneling effective mass, Φ_it_ is the electron affinity at the AlGaN/GaN interface, τ_0_ is the tunneling time constant, n is the free carrier density, σ is the capture cross-section, and υ_th_ is thermal velocity (2.6 × 10^7^ cm/s). Considering m_AlGaN_ = 0.2 m_e_ (m_e_ is the mass of the free electron) and Φ_it_ = 2.5 eV at AlGaN composition of 25 %, α_t_ is calculated to be 7.3 × 10^7^ cm^−1^ [20,21]. Assuming the dominance of carrier trapping/detrapping through the tunneling process at AlGaN/GaN heterointerface around V_th_, the value of τ_0_ is estimated to be 1 × 10^−6^ s for slow trap [22]. Therefore, the extracted tunneling depth Z is around 1.32 nm in AlGaN barrier layer at f = 10 Hz, which is smaller than that of the previous report in the partially recessed AlGaN/GaN MOSHEMT [18]. As shown in Figure 5e, the trap density N_t_ according to the trap depth (Z) for all devices is derived from the noise spectrum at V_g_~V_th_ using Equations (2) and (3). It is noted that the application of cap layer decreases the trap density excepting to the almost same N_t_ values at f = 10 Hz. The large discrepancy of trap density between three devices close to the distance from heterointerface indicates that the SiCN cap layer effectively reduces the trap density and thus well passivates the AlGaN surface.

## 4. Conclusions

We report to the fabrication and characterization of AlGaN/GaN HEMTs with (1) in situ SiCN, (2) in situ SiN, and (3) no cap layer. The in situ SiCN capped device exhibits much improved DC performances than the other devices because of the reduced positive ionic charges and enhanced surface morphology caused by C surfactant atoms during the growth of the SiCN cap layer. The pulsed I_d_−V_d_ characteristics and noise behaviors for two devices with in situ cap layers are much superior to those of the reference device without the cap layer. The obtained results tell that the in situ cap layer effectively passivates the AlGaN surface.

## Data Availability

The data is available on reasonable request from the corresponding author.

## References

[1] Kahn M.A., Chen Q., Yang J.W., Shur M.S., Dermott B.T., Higgins J.A. (1996). Microwave operation of GaN/AlGaN-doped channel heterostructure field effect transistors. IEEE Electron Device Lett..

[2] Wu Y.F., Kapolnek D., Ibbetson J.P., Parikh P., Keller B.P., Mishra U.K. (2001). Very-high power density AlGaN/GaN HEMTs. IEEE Trans. Electron. Devices.

[3] Binari S., Ikossi K., Roussos J.A., Kruppa W., Park D., Dietrich H.B., Koleske D.D., Wickenden A.E., Henry R.L. (2001). Trapping effects and microwave power performance in AlGaN/GaN HEMTs. IEEE Trans. Electron. Devices.

[4] Vetury R., Zhang N.Q., Keller S., Mishra U.K. (2001). The impact of surface states on the DC and RF characteristics of AlGaN/GaN HFETs. IEEE Trans. Electron. Devices.

[5] Luo B., Mehandru R.J., Kim J., Ren F., Gila B.P., Onstine A.H., Abernathy C.R., Pearton S.J., Fitch R., Gillespie J. (2002). Comparison of Surface Passivation Films for Reduction of Current Collapse in AlGaN/GaN High Electron Mobility Transistors. J. Electrochem. Soc..

[6] Ma J., Lu X., Jiang H., Liu C., Lau K.M. (2014). In situ growth of SiNx as gate dielectric and surface passivation for AlN/GaN heterostructures by metalorganic chemical vapor deposition. Appl. Phys. Exp..

[7] Jiang H., Liu C., Chen Y., Lu X., Tang C.W., Lau K.M. (2017). Investigation of In Situ SiN as Gate Dielectric and Surface Passivation for GaN MISHEMTs. IEEE Trans. Electron. Devices.

[8] Lee J.-H., Jeong J.-H., Lee J.-H. (2012). Enhanced Electrical Characteristics of AlGaN-Based SBD with in Situ Deposited Silicon Carbon Nitride Cap Layer. IEEE Electron Device Lett..

[9] Lee J.-H., Im K.-S., Lee J.-H. (2021). Effect of In-situ Silicon Carbon Nitride (SiCN) Cap Layer on Performances of AlGaN/GaN MISHFETs. IEEE J. Electron. Devices Soc..

[10] Choi Y.-J., Lee J.-H., Choi J.-S., An S.-J., Hwang Y.-M., Roh J.-S., Im K.-S. (2021). Improved Noise and Device Performances of AlGaN/GaN HEMTs with In Situ Silicon Carbon Nitride (SiCN) Cap Layer. Crystals.

[11] Onojima N., Hirose N., Mimura T., Matsui T. (2008). Effects of Si deposition on AlGaN barrier surfaces in GaN heterostructure field-effect transistors. Appl. Phys. Exp..

[12] Hashizume T., Ootomo S., Oyama S., Konishi M., Hasegawa H. (2001). Chemistry and electrical properties of surfaces of GaN and GaN/AlGaN heterostructures. J. Vac. Sci. Technol. B.

[13] Narang K., Bag R.K., Singh V.K., Pandey A., Saini S.K., Khan R., Arora A., Padmavati M.V.G., Tyagi R., Singh R. (2020). Improvement in surface morphology and 2DEG properties of AlGaN/GaN HEMT. J. Alloys Compd..

[14] Chroboczek J.A., Piantino G. (2000). Low Noise Current Amplifier with Programmable Gain and Polarization for Use in Electrical Measurement of Semiconductor Circuits, such as Transistors, with the Circuit Being Low Noise and Having a Protection Circuit for the Input. France Patent No..

[15] McWhorter A.L. (1957). 1/f Noise and Germanium Surface Properties in Semiconductor Surface Physics.

[16] Ghibaudo G., Roux O., Nguyen-duc C., Balestra F., Brini J. (1991). Improved analysis of low frequency noise in field-effect MOS transistors. Phys. Status Solidi A.

[17] Ghibaudo G., Boutchacha T. (2002). Electrical noise and RTS fluctuations in advanced CMOS devices. Microelectron. Rel..

[18] Takakura K., Putcha V., Simoen E., Alian A.R., Peralagu U., Waldron N., Parvais B., Collaert N. (2020). Low-Frequency Noise Investigation of GaN/AlGaN Metal–Oxide–Semiconductor High-Electron-Mobility Field-Effect Transistor with Different Gate Length and Orientation. IEEE Trans. Electron. Devices.

[19] Simoen E., Lin D.H.-C., Alian A., Brammertz G., Merckling C., Mitard J., Claeys C. (2013). Border traps in Ge/III-V channel devices: Analysis and reliability aspects. IEEE Trans. Device Mater. Rel..

[20] Grabowski S.P., Schneider M., Nienhaus H., Mönch W., Dimitrov R., Ambacher O., Stutzmann M. (2001). Electron affinity of AlxGa1−xN(0001) surfaces. Appl. Phys. Lett..

[21] Kurakin A.M., Vitusevich S.A., Danylyuk S.V., Hardtdegen H., Klein N., Bougrioua Z., Naumov A.V., Belyaev A.E. (2009). Quantum confinement effect on the effective mass in two-dimensional electron gas of AlGaN/GaN heterostructures. J. Appl. Phys..

[22] Kumar S., Gupta P., Guiney I., Humphreys C.J., Raghavan S., Muralidharan R., Nath D.N. (2017). Temperature and Bias Dependent Trap Capture Cross Section in AlGaN/GaN HEMT on 6-in Silicon With Carbon-Doped Buffer. IEEE Trans. Electron. Devices.

